# Engineering microalgae for water phosphorus recovery to close the phosphorus cycle

**DOI:** 10.1111/pbi.14040

**Published:** 2023-03-15

**Authors:** Long Wang, Xianqing Jia, Lei Xu, Jiahong Yu, Suna Ren, Yujie Yang, Kaibin Wang, Damar López‐Arredondo, Luis Herrera‐Estrella, Hans Lambers, Keke Yi

**Affiliations:** ^1^ State Key Laboratory of Efficient Utilization of Arid and Semi‐arid Arable Land in Northern China Institute of Agricultural Resources and Regional Planning Chinese Academy of Agricultural Sciences Beijing China; ^2^ Trendbiotech Co., Ltd Hangzhou China; ^3^ Department of Plant and Soil Science, Institute of Genomics for Crop Abiotic Stress Tolerance Texas Tech University Lubbock TX USA; ^4^ Laboratorio Nacional de Genómica para la Biodiversidad (UGA) Centro de Investigación y de Estudios Avanzados del IPN Irapuato, Guanajuato Mexico; ^5^ School of Biological Sciences The University of Western Australia Perth WA Australia

**Keywords:** phosphorus recovery, phosphorus removal, wastewater treatment, microalgae, genetic engineering, algal fertilizer

## Abstract

As a finite and non‐renewable resource, phosphorus (P) is essential to all life and crucial for crop growth and food production. The boosted agricultural use and associated loss of P to the aquatic environment are increasing environmental pollution, harming ecosystems, and threatening future global food security. Thus, recovering and reusing P from water bodies is urgently needed to close the P cycle. As a natural, eco‐friendly, and sustainable reclamation strategy, microalgae‐based biological P recovery is considered a promising solution. However, the low P‐accumulation capacity and P‐removal efficiency of algal bioreactors restrict its application. Herein, it is demonstrated that manipulating genes involved in cellular P accumulation and signalling could triple the *Chlamydomonas* P‐storage capacity to ~7% of dry biomass, which is the highest P concentration in plants to date. Furthermore, the engineered algae could recover P from wastewater almost three times faster than the unengineered one, which could be directly used as a P fertilizer. Thus, engineering genes involved in cellular P accumulation and signalling in microalgae could be a promising strategy to enhance P uptake and accumulation, which have the potential to accelerate the application of algae for P recovery from the water body and closing the P cycle.

## Introduction

Being a finite and non‐renewable resource, phosphorus (P) is primarily mined from rock P reserves, which are limited to a small number of geographical regions (Cordell *et al*., 2009; MacDonald *et al*., 2011), and about 82% of P rock extracted globally is for P fertilizers (Cordell and White, 2014). Off‐site release of P due to anthropogenic activity increases environmental pollution, including release from industrial wastewater, municipal sewage effluent, and agricultural run‐off (Cordell and White, 2014). P loss from agriculture contributed more than 38% of the P load in freshwater systems (Mekonnen and Hoekstra, 2018), and about 30–50% of P in P fertilizers is exported via wind and water erosion into water bodies (Cordell and White, 2014; Liu *et al*., 2016). Increasing P release into the environment, the rate‐limiting nutrient for phytoplankton growth, has promoted toxic algae blooms that seriously damage livings in aquatic ecosystems (Elser and Bennett, 2011; Schindler *et al*., 2016). Thus, reducing off‐site P release to adjacent ecosystems is key to reducing the eutrophication (Schindler *et al*., 2016). Due to extensive P mining, massive loss of P sources in agricultural and industrial production, and a lack of efficient P‐recycling approaches, the broken P cycle is threatening the ecosystems and global food security (Elser and Bennett, 2011; Gilbert, 2009; Withers, 2019); thus, closing the P cycle is of increasing concern and appeal (Cong *et al*., 2020).

To ensure that P resources are adequate for food security and to reduce environmental pollution, sustainable approaches to P recovery from waste and recycling it for use in agriculture are required. As an environmentally friendly and sustainable alternative to energy‐intensive and conventional biological treatment processes, enhanced biological P removal (EBPR) is increasingly used in P recovery from wastewater (Li *et al*., 2019a; Solovchenko *et al*., 2016; Xu *et al*., 2020). EBPR is usually executed by polyphosphate (polyP)‐accumulating organisms (PAO), commonly bacteria and algae. Bacteria‐based EBPR systems have been used extensively to remove nutrients from wastewater, but it usually causes a large carbon and N_2_O emission, and the recovered P is often difficult to reuse easily (Li *et al*., 2019a). Like bacteria, algae preferentially absorb and utilize P as inorganic phosphate (Pi), and excess Pi is stored as polyP in algal vacuoles (also called acidocalcisomes) (Aksoy *et al*., 2014). Algae can perform sustained luxury P uptake (i.e., they take up more P than required for their immediate growth), grow fast, use nutrients in wastewater, and produce biomass suitable for algae‐based fertilizer production. Thus, given the low operational cost and avoidance of sludge‐handling problems, algae‐based EBPR systems offer attractive nutrient‐removal alternatives (Li *et al*., 2019a).

Recently, improved algae‐based EBPR systems that incorporate membrane bioreactors (Qin *et al*., 2020) or optimize processes (Abeysiriwardana‐Arachchige *et al*., 2021) were proposed. However, two main issues limit the development of the algae‐based EBPR system (Li *et al*., 2019a; Solovchenko *et al*., 2016; Xu *et al*., 2020): (1) compared with bacteria‐based EBPR systems, its P‐removal efficiency still needs to be improved (summarized in Table S1); and (2) although the total P concentration in algae is much greater than that in land plants, ranging from 10–30 mg/g dry matter (Procházková *et al*., 2014) (as measured and shown in Table S2), increasing their P‐accumulation capacity is still warranted.

The recent discoveries of the major transcription factor for P signalling in the model green alga *Chlamydomonas reinhardtii* and its tonoplast‐located P‐efflux transporter have enhanced the understanding of the cellular P accumulation and signalling mechanisms in algae. These findings showed that P‐deficiency responses are regulated mainly by the MYB transcription factor *Phosphate Starvation‐Responsive 1* (*PSR1*) in *Chlamydomonas reinhardtii* (Moseley *et al*., 2006; Wykoff *et al*., 1999), and loss‐of‐function of *PSR1* caused compromised P signalling and a reduced total cellular P concentration (Bajhaiya *et al*., 2016; Wang *et al*., 2020). Moreover, the loss‐of‐function of a tonoplast‐located Pi‐efflux transporter, Phosphate Transporter C1 (CrPTC1), an SPX‐SLC protein with both SPX (named after SYG1/Pho81/XPR1) and SLC (named after permease solute carrier 13) domains causes excess polyP accumulation in vacuoles and strongly induces P‐starvation response in *C. reinhardtii* (Wang *et al*., 2021).

To improve the P‐accumulation capacity and P‐removal efficiency of algal bioreactors and further accelerate the application of algae for water treatment to recover P and tighten the P cycle, herein, engineering genes involved in cellular P accumulation and signalling in *C. reinhardtii* is reported to generate modified algae with greater luxury P uptake and greater P‐accumulation capacity. The engineering strategy is based on knocking out the *PTC1* gene and overexpressing the *PSR1* gene, respectively, alone or in combination. Engineered strains were further assessed and employed to recover P from artificial and industrial wastewater. All engineered strains accumulated more P and showed a greater ability to remove P without compromising biomass production than the wild‐type (WT). These approaches have the potential to accelerate the application of algae for water treatment to recover P as algal fertilizer and tighten the P cycle.

## Results

### Entrapping polyP into vacuoles enhances P removal

**Figure 1:**
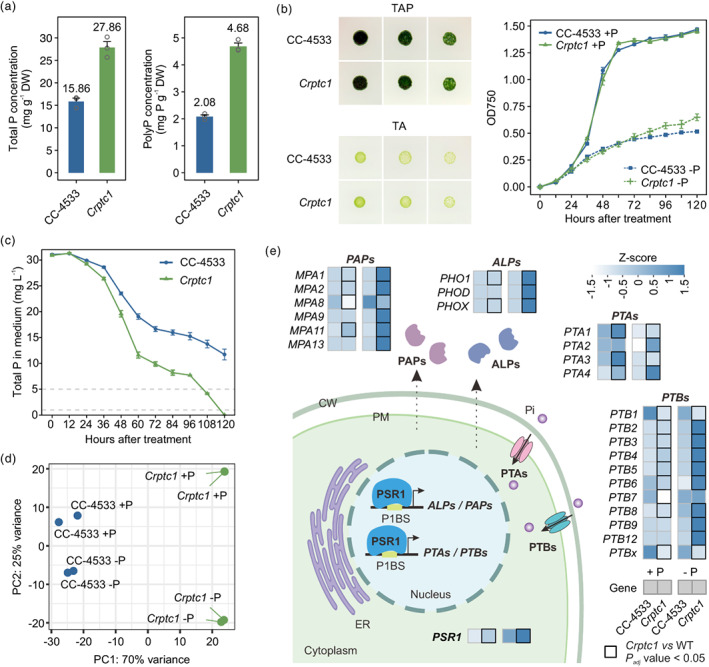
Knockout of *CrPTC1* confers high phosphorus (P) removal capacity without compromising cell growth. (a) Total P and polyP concentrations of CC‐4533 and the *Crptc1* mutant. Error bars indicate SE. (b) Growth of CC‐4533 and the *Crptc1* mutant strain in the TAP (with Pi supply) and TA (without Pi supply) media. Colonies from left to right depict a series of dilutions. The panel at the right shows the growth curves of CC‐4533 and the *Crptc1* mutant under Pi‐supply (+P) and Pi‐deprivation (−P) conditions. Error bars indicate SE. (c) Assessment of P‐removal ability of CC‐4533 and the *Crptc1* mutant with 31 mg/L P supply. Grey lines on 1 and 5 mg/L show the first‐level and third‐level water quality threshold values, respectively, according to the discharge standard of pollutants for municipal wastewater treatment plants in China (GB18918‐2002). Error bars indicate SE. (d) Principal component analysis (PCA) shows the global similarity and divergence of transcriptome data. The first two components are shown in the plot. (e) Heatmap of expression profiles of genes involved in P accumulation under +P and −P conditions. ALPs, alkaline phosphatases; PAPs, purple acid phosphatases; PTA, phosphate transport family A; PTB, phosphate transport family B. CW, cell wall; PM, plasma membrane.

Given that algae can perform sustained luxury P uptake and store excess Pi as polyP in vacuoles, it is hypothesized that engineering the tonoplast‐located P transporters will lead to increased accumulation of polyP in vacuoles and thus further enhance luxury P uptake. In the previous study, it is shown that CrPTC1 is involved in the efflux of vacuolar Pi, and that the loss‐of‐function of *CrPTC1* causes greater P and polyP accumulations in vacuoles (Wang *et al*., 2021). Total P and polyP concentrations in the *Crptc1* mutant were around two‐fold higher than those in WT (Figure 1a), suggesting that the *Crptc1* mutant has a higher P‐removal capacity than the WT. In addition, in the design of engineered microalgae, an efficient PAO is expected to have a high P‐removal capacity without compromising cell viability under either P‐sufficient or P‐deficient conditions (Xu *et al*., 2020). To test this, the growth status of the *Crptc1* mutant was evaluated under both Pi‐sufficient and Pi‐deficient conditions (Figure 1b). Like the WT (CC‐4533), the *Crptc1* mutant grew less in P‐depleted conditions than in P‐replete conditions, but its growth was not significantly different from the WT in either condition (Figure 1b). These data suggest that the *Crptc1* mutant has the potential to be used as an improved PAO for P removal from wastewater. Then the P‐removal capacity of the *Crptc1* mutant was assessed by simulating the wastewater environment through an external 31 mg L^−1^ P supply (Figure 1c). After 120 h (5 days), the *Crptc1* mutant had removed nearly all Pi from the medium, while the WT only removed around 62% P, leaving a final P concentration of 11.7 mg/L in the medium. It took about 216 h (9 days) for the WT to remove all P from the medium (Figure S1). These results confirmed that *Crptc1* has a great capacity of P removal.

To gain insights into the possible mechanism enabling *Crptc1* to remove Pi more efficiently than WT did, gene‐expression profiles of the *Crptc1* mutant were analysed under Pi‐sufficient conditions and after 6 h of Pi starvation using RNA‐seq. Principal component analysis (PCA) shows the global similarity of bioreplicates for each treatment and significant differences in expression profiles between the *Crptc1* mutant and CC‐4533 under both Pi‐sufficient (+P) and Pi‐deficient (−P) conditions (Figure 1d). Gene ontology (GO) enrichment analysis of significantly up‐regulated genes in the *Crptc1* mutant under P starvation shows that terms related to ion transport were enriched considerably (Figure S2). Among them, the term with the highest gene ratio was enriched in phosphate ion transport (GO:0006817 and GO:0005315). Notably, a large set of genes known to be involved in Pi signalling and accumulation were strongly up‐regulated in the mutant respect to the WT in both +P and −P conditions and particularly more in the −P condition. This gene set included the core regulator *PSR1* and genes from several Pi‐regulation‐related gene families, such as alkaline phosphatase (ALP), purple acid phosphatase (PAP), Pi transport A (PTA), and B (PTB) families (Figure 1e). Taken together, these results indicate that loss‐of‐function of CrPTC1 caused a greater accumulation of polyP in vacuoles and disrupted the cellular P regulation, which thus caused a more sensitive Pi‐starvation signalling and further enhanced Pi uptake. These results suggest that increasing the expression of the core regulator *PSR1* may also be a strategy to increase P removal in algae.

### Enhanced Pi signalling improves P removal

**Figure 2:**
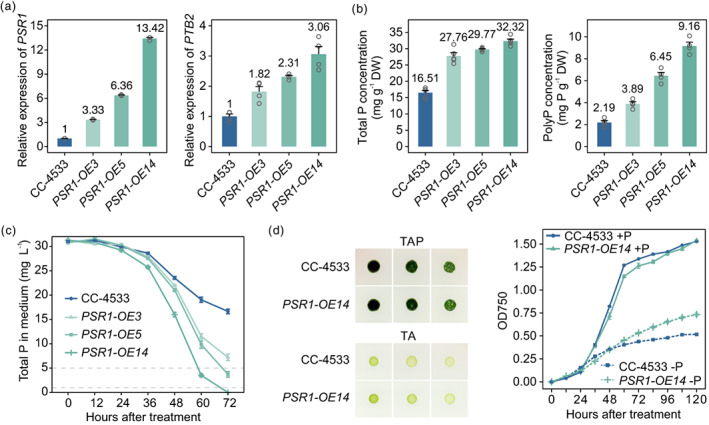
Over‐expression of *PSR1* conferred high phosphorus (P) removal capacity. (a) Relative expression levels of *PSR1* and *PTB2* in three representative *PSR1‐OE* lines. Error bars indicate SE. (b) Total P and polyP concentrations in the *PSR1‐OE* lines. (c) P‐removal ability of the *PSR1‐OE* lines with 31 mg/L P supply. (d) Growth of CC‐4533 and the *PSR1‐OE14* lines in the TAP and TA media. Colonies from left to right represent a series of dilutions. The panel at the right shows the growth curves of CC‐4533 and the *PSR1‐OE14* line under 31 mg/L P‐supply (+P) and Pi‐deprivation (−P) conditions.

To test if over‐expression of *PSR1* increases P removal, *PSR1* over‐expression (*PSR1‐OE*) lines with different expression levels of *PSR1* were developed (Figure 2a) and their growth characteristics and P‐recovery potential were further evaluated. *PSR1* expression level was determined in three *PSR1‐OE* lines, which showed a three‐, six‐, and 13‐fold greater expression of *PSR1* than that of the WT. The relative expression of the Pi transporter – *PTB2* was also measured, which was also greater in all *PSR1‐OE* lines (Figure 2a). These findings indicate greater P uptake in the *PSR1‐OE* lines than in the WT. As expected, both total P and polyP showed significantly higher concentrations in all three *PSR1‐OE* lines (Figure 2b). Further P‐removal recreation experiments demonstrated that all *PSR1‐OE* lines had excellent P‐removal capacity (Figure 2c), and more importantly that the greater the *PSR1* expression level in overexpression lines, the shorter the time required for complete P removal. The *PSR1‐OE14* strain with the greatest expression of *PSR1* completely removed P after 72 h (3 days), and was selected for further analyses. Growth assessment showed no apparent loss of fitness in *PSR1‐OE14* under either +P or −P conditions compared with the WT (Figure 2d). Given the above, increasing the expression of *PSR1* would also increase P removal.

### Combining enhancing Pi signalling and entrapping P in vacuoles further enhances P removal

**Figure 3:**
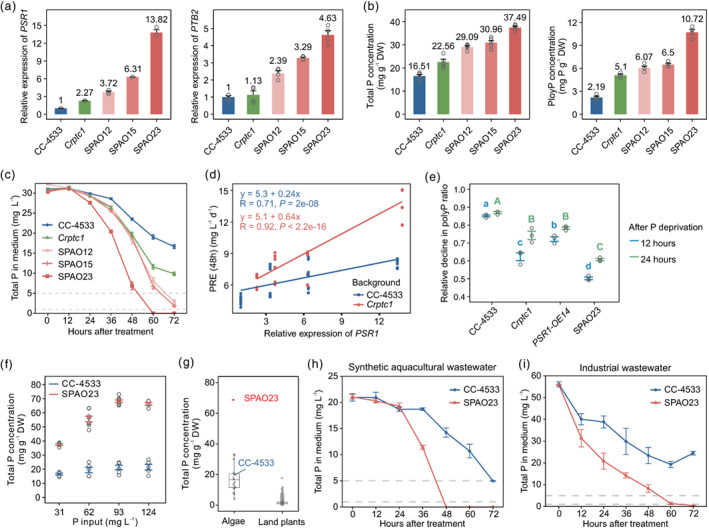
Over‐expression of *PSR1* in the *Crptc1* mutant further enhanced phosphorus (P) removal. (a) Relative expression levels of *PSR1* and *PTB2* of three representative SPAO lines. (b) Total P and polyP concentrations in the SPAO lines. (c) Assessment of the P‐removal capacity of the SPAO lines with a 31 mg/L P supply. (d) Correlation of P‐removal rate (PRR) and relative expression of *PSR1* under backgrounds of CC‐4533 (blue) and the *Crptc1* mutant (red). PRR results were calculated at 48 h with a 31 mg/L P supply. (e) The relative decline in the polyP ratio of different strains under P deprivation. Differences were tested by ANOVA using the LSD method with a Bonferroni correction at α = 0.05. Letters with the same colour (blue or green) refer to the test for the same group. Means with the same letters were not significantly different. (f) Total P concentrations of CC‐4533 and SPAO23 strains cultured in the medium with different P inputs. Error bars indicate SE. (g) Total P concentration of land plants and algae. Data on the total P concentration of land plants were collected from the previous study (Reich and Oleksyn, 2004) and from measurements in this study (Table S2). (h–i) Evaluation of P‐removal capacity of the CC‐4533 and SPAO23 lines with synthetic aquacultural wastewater (SAWW) (h) and with actual industrial wastewater (i). Error bars indicate SE.

The above results showed that both inducing Pi‐starvation signalling by increasing *PSR1* expression and knockout of *CrPTC1* to accumulate polyP in vacuoles increased luxury P uptake. Thus, it is hypothesized that combining these two approaches could further enhance the P‐removal capacity. To test this, *PSR1* was then overexpressed in the *Crptc1* mutant background. Three *PSR1‐OE:Crptc1* lines were chosen for further characterization, which presented a greater expression of *PSR1* than that of the WT, as well as a greater expression of *PTB2* (Figure 3a). Also, both total P and polyP showed significantly higher concentrations in all three *PSR1‐OE*:*Crptc1* lines than in the WT, and strains with greater *PSR1* expression showed more accumulations of total P and polyP (Figure 3b). These engineered lines ‘super (polyP)‐accumulating organism’ (SPAO) were thus termed. Notably, strains in the *Crptc1* mutant background showed greater P accumulation than that in the WT background with similar *PSR1* expression levels, for example, SPAO23 (relative *PSR1* expression, 13.82; total P, 37.49 mg/g DW) (Figure 3a,b) and *PSR1‐OE14* (relative *PSR1* expression, 13.42; total P, 32.32 mg/g DW) (Figure 2a,b). Besides P, SPAO23 also over‐accumulated Ca and Mg in cells (Table S3), which is probably the consequences of intracellular ion homeostasis.

P‐removal evaluations showed that all SPAO lines had superior P‐removal capacities than the WT and *Crptc1* mutants (Figure 3c). Among them, the SPAO23 line completely removed P in the medium after 60 h, much faster than both the *PSR1‐OE14* line and *Crptc1*, which completely removed P after 72 h (Figure 2c) and 120 h (Figure 1c), respectively. Growth measurements showed no growth defects in the SPAO23 line, irrespective of Pi sufficiency or Pi deficiency (Figure S3). It is found that there is a direct correlation between PRR (48 h) (P‐removal rate after 48 h treatment, defined in Methods) and *PSR1* expression both in the WT background (*R* = 0.71, *P* value = 2 × 10^−8^) and the *Crptc1* mutant background (*R* = 0.92, *P* value <2.2 × 10^−16^) (Figure 3d). The strains in the *Crptc1* background with similar *PSR1* expression levels had a much higher PRR (slope in the *Crptc1* background vs. WT, 0.62 vs. 0.24; *P* value = 1.36 × 10^−6^, *t*‐test) (Figure 3d). These suggested that trapping P in vacuoles significantly contributes to enhancing PRR, while the combination of the two approaches offered even better results.

To further confirm the contributions of trapping P in vacuoles, polyP staining and quantification were performed before and after P starvation in four representative strains, including CC‐4533, *Crptc1*, *PSR1‐OE14*, and SPAO23. After 48 h of sufficient P supply, the polyP contained in vacuoles was: SPAO23 > *PSR1‐OE14* > *Crptc1* > WT (Figure 3e and Figure S4), consistent with the polyP concentrations measured above (mg P g^−1^ DW, 10.7 > 9.2 > 5.1 > 2.2). However, after 24 h of P starvation, the polyP contained in vacuoles was: SPAO23 > *Crptc1* > *PSR1‐OE14* > WT. Specifically, after Pi starvation, SPAO23 still contained the most polyP in vacuoles, but the *Crptc1* mutant had more polyP accumulated in vacuoles than *PSR1‐OE14*. After 12 h of P starvation, WT and *PSR1‐OE14* strains, respectively, showed ~85% and ~72% relative declines in polyP ratio, but the *Crptc1* mutant and SPAO strains retained more polyP in vacuoles, with 62% and 50% decline in polyP ratio, respectively. Similar decline trends were observed after 24 h of P starvation. Specifically, *PSR1‐OE* and WT lines showed a more substantial reduction in polyP concentration than SPAO23 and *Crptc1*. These results indicated that *PSR1‐OE* and WT lines did not continue trapping P in vacuoles when P in solution gradually decreased, because P stored in vacuoles was remobilized under P deficiency to meet the cellular P requirement (Yang *et al*., 2017). Thus, it is proposed that trapping P in vacuoles via engineering vacuolar P transporter is vital for maximizing P removal.

Given that the SPAO23 strain accumulated more than twice as much total P as the WT did under normal culture conditions (Figure 3b), then, it is asked what is the possible maximum P‐accumulation capacity in SPAO strains? To assess this, the SPAO23 strain and the WT were cultured in modified TAP conditions amended with different concentration of Pi. Total P concentrations increased with increasing P input and did no further increase after P input exceeded 93 mg/L (3 times of the normal concentration in TAP) (Figure 3f). At 93 mg/L P input, the total P concentration in the SPAO23 line reached a maximum of 68.3 mg/g DW, while the total P concentration in WT was relatively stable at about 20 mg/g DW (Figure 3f). Thus, it is proposed that the over‐expression of *PSR1* in the *Crptc1* background can enormously increase maximum P‐accumulation capacity up to almost 7% dry matter. So far, this is the maximum stoichiometric proportion of total P that has to be achieved in plants (Figure 3g and Table S2).

Taken together, these data suggest that the over‐expression of *PSR1* in the *Crptc1* background, namely, combining enhancing Pi signalling and entrapping P in vacuoles, can further enhance P‐removal rate.

### Removing P from artificial and industrial wastewater

To evaluate the performance of SPAO strains in wastewater treatment, simulated evaluations of the SPAO strains were conducted with synthetic aquacultural wastewater (SAWW). Aquaculture wastewater is usually considered to be the leading cause of harmful algal blooms of watercourses and changes in aquatic biota (de Vasconcelos *et al*., 2021), and the loss of dietary P in aquaculture is up to 60% (Sugiura, 2018). Aquaculture wastewater always shows a higher nitrogen‐to−Phosphorus ratio (N/P ratio) (de Vasconcelos *et al*., 2021). Here, a SAWW with a higher N/P ratio than the common growth condition (10.5:1 vs. 7:1) was set up. With about 20 mg/L total P input and an approximately 1% initial inoculum (about 10^5^ cells/L), SPAO23 completely removed P after 48 h (2 days) in the SAWW, whereas the WT strain only removed 75% of total P after 72 h (3 days) (Figure 3h). These results showed that SPAO strains have strong application potential in the treatment of wastewater with a high N/P ratio.

To evaluate the P‐removal capacity of the engineered algae strains in the real wastewater environment, the wastewater from a chemical plant in Nantong, China, was collected for further analysis. The wastewater was used in the experiments directly without filtration to minimize any change in water composition. Characteristic analysis showed that the wastewater contained 56 mg/L total P and 34 mg/L total N, with 1100 mg/L chemical oxygen demand (COD). To simulate the actual wastewater treatment scenario as much as possible (Nie *et al*., 2020), after adjusting the wastewater to the algal growth conditions (details in Methods), an approximately 10% initial inoculum (about 10^6^ cells/L) of SPAO23 strains and its WT were used to inoculate wastewater in a 50 mL working volume. Measurement of the residual P in the wastewater showed that SPAO23 had removed 97.4% of the total P from the wastewater after 60 h, and it recovered all the P within 72 h (Figure 3i). In contrast, the wastewater inoculated with the WT strain reached the lowest residual P at 60 h (34.5% of initial P concentration), and this even increased with prolonged cultivation (Figure 3i). The above results confirmed that SPAO23 has superior P‐removal application prospects.

Together, these results show that the SPAO23 strain had a greater P‐removal capacity than WT did, both in the simulated conditions and in the treatment of industrial wastewater.

### Potential as P fertilizer

All the engineered strains exhibited a substantial accumulation of total P and polyP, especially the SPAO strains. The next step is to define the best use of this P‐enriched algal biomass. Algal fertilizer is increasingly being proposed as an environmentally friendly bio‐based fertilizer for pollution‐free agricultural applications, owing to its tremendous potential to immobilize nutrients and make them bioavailable through the controlled release of nutrients (Win *et al*., 2018). The algal cells can be applied to grow together with the plants to avoid nutrient deficiencies, or manufactured as dried algal fertilizers to increase the availability of essential nutrients with a constant ratio of N/P (Nosheen *et al*., 2021). Thus, it is hypothesized that algae used to treat wastewater could be as effective as chemical fertilizers in improving soil fertility and increasing crop productivity. SPAO strains with the greatest P accumulation were expected to have a greater potential as environmentally friendly algal fertilizers, especially for P supply. Here, to simplify the comparison, P was set as the only limiting nutrient for plant growth by supplying enough of all other elements (without P) and planting tomato plants in a synthetic low‐P matrix. Pi, the only form taken up by plants, was used as the control chemical P fertilizer treatment, and applied algal cells to provide with the same Pi input for plant growth as the chemical fertilizer. After 30 days of growth, compared with significantly more sluggish growth of plants lacking P supply, plants fertilized with Pi and algal cells showed a clear growth advantage, and plants treated with SPAO23 showed greater biomass than plants treated with the same amount of the WT strain (Figure S5). Taken together, these results highlight the fertilizer potential of algae used for wastewater treatment and suggest that algae with greater polyP accumulation can be used as a slow‐release P fertilizer.

## Discussion

With increasing urbanization and the demand for agricultural production, increasingly more P is lost to the environment through effluent discharge, and P recovery is urgently needed to tighten the P cycle to ensure food security and reduce environmental pollution (Elser and Bennett, 2011; Gilbert, 2009; Peñuelas and Sardans, 2022). As an environmentally friendly and sustainable alternative, P removal by algae or their aggregates is increasingly employed in wastewater treatment (Mohsenpour *et al*., 2021). Much progress has been made in previous studies to maximize P recovery of algae‐based EBPR systems, including coordinating algae growth conditions that affect P‐removal efficiency (Powell *et al*., 2009), enhancing P entrapment and resistance to environmental stress by microalgae–bacteria interactions (Xu *et al*., 2020), and integrating with algal biofilm reactors or immobilized‐algae EPBR designs (Solovchenko *et al*., 2016). However, the relatively low P‐removal efficiency in current algae‐based EPBR systems limits its potential for a widely used cycle that takes P removal from waste streams to crop plant fertilizer and algal strains with a high capacity of taking up and storing large quantities of P in their cells are anticipated (Nie *et al*., 2020; Solovchenko *et al*., 2016). In addition, the area required by P‐recovery plants depends on their P content and areal biomass production, and P recovery by algae with high P concentration was proposed as the most area‐saving and P‐recycling strategy compared with recovery by other aquatic plants (Shilton *et al*., 2012).

In this study, by increasing *PSR1* expression under the *Crptc1* background, the P concentration of the algal biomass could be increased more than doubled, up to 6.83%, which is comparable to the stoichiometric proportion of total P in microbes (about 5–10%) (Yao *et al*., 2016; Zhang and Elser, 2017). Thus, it is indicated that engineering genes involved in P accumulation and signalling to enhance luxury P uptake and accumulation in algae *per se* would be a fundamental and promising approach for improving algae‐based EBPR systems.

**Figure 4:**
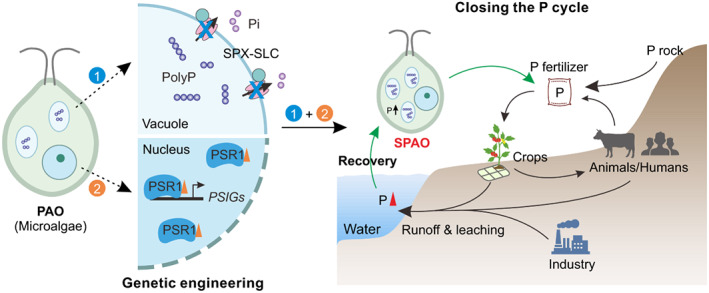
The proposed model for SPAO design and closing the P cycle. The proposed model for SPAO design is shown on the left. Compared with conventional PAO (wild‐type microalgae), SPAO presents greater polyP accumulation and higher P‐removal capacity. Three approaches for genetic engineering of SPAO to enhance the P‐removal capacity are suggested: (1) genetic operation of genes controlling the vacuolar P accumulation. Down‐regulation (or loss‐of‐function) of SPX‐SLC proteins to enhance P accumulation in vacuoles and further increase the P‐removal capacity in SPAO; (2) increase the expression of PSR1, which enhances Pi acquisition through directly up‐regulating the expression of P‐starvation‐induced genes (PSIGs); (3) combining the above two approaches – enhancing P‐starvation signalling and accumulating P in vacuoles. After being applied to recover P from the water system, SPAO algae with greater polyP accumulation can be used as a slow‐release P fertilizer to close the P cycle.

Here, three improved approaches involving genetic engineering of SPAO were proposed (Figure 4): (1) genetic operation of genes controlling vacuolar P accumulation. Down‐regulation (or loss‐of‐function) of SPX‐SLC proteins to enhance P and polyP accumulation in vacuoles and further increase the P‐removal capacity in SPAO; (2) increasing the expression of the core regulator of the Pi‐starvation response – PSR1 to further enhance Pi acquisition through directly up‐regulating the expression of P‐starvation‐induced genes (PSIGs), including Pi transporters that are responsible for Pi absorption from the environment, and ALPs which liberate Pi from dissolved organic P compounds; (3) combining the above two approaches – enhancing Pi‐starvation signalling and accumulating P in vacuoles. Based on assessment in *Chlamydomonas* in this study, the engineered SPAOs through the third approach enhance the P‐removal capacity from artificial wastewater to almost three times that of WT (complete P‐removal time for SPAO23 vs. WT: 60 h vs. 216 h).

Species of several algal genera have been assessed and employed for P removal from wastewater, such as *Chlorella*, *Scenedesmus*, *Oocystis*, and *Ankistrodesmus* (Mohsenpour *et al*., 2021). In this study, it is shown that engineering the genes involved in the Pi signalling and accumulation could enhance the luxury P uptake and enable the development of species or strains that are more efficient at P removal from wastewater. PSR1 is conserved to regulate the Pi‐starvation signalling in green plants and algae (Jia *et al*., 2021, 2023; Rubio *et al*., 2001). The previous study also demonstrated that SPX‐SLC proteins are widely found in green algae and responsible for the P accumulation in green algae (Wang *et al*., 2021). Specifically, these conservative mechanisms of Pi signalling and accumulation are widespread in green algae. Thus, although this study takes the model green alga *C. reinhardtii* as an example, it is possible to broadly produce genetically engineered microalgae to enhance their ability to remove P from wastewater.

In summary, this study reported the genetic engineering approaches in microalgae that can increase the concentrations of total P more than three times in algal cells, which made P‐removal capacity from wastewater more efficient as the engineered strains removed P three times quicker than the unengineered algae did. Given the conservation of PSR1 and SPX‐SLC proteins in green algae (Jia *et al*., 2021; Rubio *et al*., 2001; Wang *et al*., 2021), the approach reported in this study is likely to function in other microalgae species for wastewater treatment to recover P as algal fertilizer and tighten the P cycle.

## Experimental procedures

### *Chlamydomonas reinhardtii* strains and growth conditions

The *Chlamydomonas reinhardtii* strain CC‐4533 (also called CMJ030) and *Crptc1* (LMJ.RY0402.181899) were purchased from the *Chlamydomonas* Resource Center (*Li et al*., 2019b). Cells were cultured in a standard Tris‐acetate‐phosphate (TAP) medium (Harris, 1989) at pH 7.0 under continuous illumination (50 μmol photons/m^2^/s) on a rotating platform (150 rpm) at 24°C. TAP medium contains: 2.42 g/L Tris, 0.375 g/L NH_4_Cl, 0.1 g/L MgSO_4_.7H_2_O, 0.05 g/L CaCl_2_. 2H_2_O, 10.5 mg/L K_2_HPO_4_, 5.4 mg/L KH_2_PO_4_, 1 mL/L glacial acetic acid, and 1 mL/L Hunter's Trace Stock Solution (Harris, 1989). For Pi deprivation, cells were pelleted by centrifugation (2000 g, 5 min), washed twice with TA, that is, TAP in which potassium chloride was substituted for potassium phosphate (Quisel *et al*., 1996), and then resuspended in the TA medium.

### Generation of *Chlamydomonas* over‐expression lines

To develop *CrPSR1* over‐expression strains, the genomic DNA of *CrPSR1* was introduced into the HSP70A‐RBCS2‐Ble vector (Zhang *et al*., 2021), then the reconstructed plasmids were linearized with ScaI before electroporation into *Chlamydomonas* cells. Transformants were selected on the solid TAP medium containing 10 μg/mL bleomycin (Shimogawara *et al*., 1998). Positive transformants were further validated by the relative expression level of *CrPSR1* using qRT‐PCR.

### Wastewater source and experimental set‐up

Industrial wastewater used in this study was collected from a chemical plant located in Nantong, China, which contains 34 mg/L total N and 56 mg/L total P, with 2200 mg/L chemical oxygen demand (COD). To adjust the wastewater to the algal growth conditions, 3 mm NH_4_Cl, 0.2 mm MgSO_4_, 0.34 mm CaCl_2_, and 0.5 mL/L Hunter's Trace Stock Solution (Harris, 1989) were added to generate experimental conditions. The pH was controlled at approximately 7.0. The working volume was 50 mL, and the initial inoculum was approximately 10% (about 10^6^ cells/L).

Synthetic aquaculture wastewater (SAWW) was prepared based on the characteristics of local aquaculture wastewater from Zhoushan, China. The components were: ammonium, 120 mg/L; orthophosphate, 20 mg/L; and 92.3 mg/L of CH_3_COONa as an additional carbon source. Other nutrients were added as the TAP medium. The pH of the SAWW was controlled at approximately 7.0. The working volume was 50 mL, and the initial inoculum was approximately 1% (about 10^5^ cells/L).

For other simulating experiments, the standard TAP medium was used. The working volume was 50 mL, and the initial inoculum was approximately 1% (about 10^5^ cells/L).

For the residual P content analysis, 0.2 mL medium was sampled every 12 h and the algae cells were removed by centrifugation.

### Measurement of polyP, Pi, and total P

For the measurement of total polyP, 0.5 mL algal cells were harvested (2300 g, 2 min), and the pellet was frozen immediately at −20°C for later analysis. After thawing, 50 μL of 1 m H_2_SO_4_ was added to the cells. The polyP was purified using PCR purification columns (Werner *et al*., 2005), thus excluding short‐chain polyPs. A 5 μL polyP solution was mixed with an equal volume of 2 m HCl and heated at 95°C for 30 min. The Pi released from polyP was measured by the molybdenum blue method as described in the previous study (86 μL of 28 mm ammonium heptamolybdate in 2.1 m H_2_SO_4_ and 64 μL of 0.76 mm malachite green in 0.35% (v/v) polyvinyl alcohol) (Zhang *et al*., 2022). The absorbance was measured at a wavelength of 595 nm in a TECAN infinite Elisa Reader. PolyP within algal cells were stained with DAPI, as described previously (Aschar‐Sobbi *et al*., 2008). For the total P concentration, 10 mL of algal cells were harvested, dried, and then digested with 65% HNO_3_ at 75°C for 6 h; total P content was analysed by the malachite green method (Cogan *et al*., 1999) and expressed per unit dry weight.

For the Pi measurement of tomato leaves, a 0.1 g fresh sample was homogenized with 0.5 mL of 5 m H_2_SO_4_ and 1 mL H_2_O, centrifuged and measured using malachite green (Cogan *et al*., 1999). For total P in the water, 5% (w/v) potassium persulfate was added to the sample and autoclaved the solution at 121°C for 30 min, and measured the total P concentration with malachite green.

### Elemental analysis

For elemental analysis, 10 mL of algal cells was harvested, dried, and then digested with 65% HNO_3_ at 75°C for 6 h. The elemental concentration was determined by inductively coupled plasma optical emission spectrometry (ICP‐OES; Thermo Scientific).

### Quantitative real‐time PCR analysis

Total RNA was extracted from frozen cell pellets using the Rneasy Mini Kit (Qiagen) and reverse transcribed to complementary DNA after Dnase I treatment following the standard instructions (NEB). Quantitative real‐time PCR was performed using an SYBR Premix kit (Roche) on QuantStudio 6 Flex equipment (Life Technologies). The *CBLP* gene was used as an internal control (Chang *et al*., 2005). The primer pairs used for RT‐qPCR are given in Table S4.

### P‐removal efficiency determination

To evaluate the P‐removal effects of algal strains, the P‐removal percentage (PRP), which means the proportion of removed P after a certain time, was defined as:

(1) $$\mathrm{PRP}=\frac{P_{\mathrm{ic}}-P_{\mathrm{fc}}}{P_{\mathrm{ic}}}\times 100$$

where *P*_ic_ and *P*_fc_ are the initial and final concentrations of total P retained in wastewater, respectively.

Then, to evaluate the overall P‐removal rate, the PRR_(f)_ (mg/L/d) was defined as:

(2) $${\mathrm{PRR}}_{\left(f\right)}=\frac{P_{\mathrm{ic}}\times \mathrm{PRP}}{t_{f}}$$

where *P*_ic_ is the initial concentration of total *P* retained in wastewater; *t*_f_ is the total time consumed at the final point.

To compare the P‐removal rate of different strains at a certain time point, the average P‐removal rate in the period *t*, PRR_(*t*)_ (mg/L/d), was defined as:

(3) $${\mathrm{PRR}}_{\left(t\right)}=\frac{P_{\mathrm{ic}}-P_{\mathrm{tc}}}{t}$$

where *P*_*ic*_ and *P*_*tc*_ are the concentrations of total P retained in wastewater at the initial point and sampled point after *t* days (or hours) of treatment, respectively.

### Tomato material and growth conditions

*Solanum lycopersicum* ZF702 plants were used for cultivation experiments. The planting experiments were carried out in a synthetic low‐P matrix of a mixture of coconut coir:perlite:vermiculite = 1:1:1, with a Pi concentration <2.5 mg/kg and a total P concentration < 10 mg/kg. To prepare algal fertilizers, algal cells were treated as follows: 100°C for 10 min, then ultrasonication for 30 min. Tomato plants were transplanted to the synthetic low‐P matrix after seed germination. Then all plants were fertilized with 10 mL H_2_O (mock), 0.5 mg P (10 mL 212 mg/L KH_2_PO_4_), 10 mL CC‐4533 cells, 5 mL SPAO23 cells, and 10 mL SPAO23 cells once a week. The total P concentrations of CC‐4533 cells and SPAO cells were about 50 mg/L and 100 mg/L, respectively. Other nutrients for each treatment were supplied with Murashige and Skoog (MS) basal medium (without P). Pi concentration and biomass of tomato shoots for each treatment were measured at 30 days after transplanting.

### RNA sequencing and data analysis

Total RNA was extracted by TaKaRa MiniBEST Universal RNA Extraction Kit, and at least two independent biological replicates were used for each line. Library construction of RNA and sequencing was carried out by HiSeq 4000 platform with paired‐end (2 × 150 bp) sequencing. Transcriptome data were prepared and analysed as described in the previous study (Zhang *et al*., 2022). Significant changes in differentially expressed genes (DEGs) were determined as fold‐change >2 and fold‐change < −2 for up‐regulation and down‐regulation, respectively, with *P*_adj_ value <0.05. Significantly enriched GO items were filtered by *P* value <0.01 and false discovery rate (FDR) < 0.05. Diagrams were drawn by R scripts available on request.

## Conflict of interest

The authors declare no conflict of interest. K.Y., L.W., and X.J. are inventors of the patent application PCT/CN2022/086874 based on this work.

## Author contributions

L.W. and X.J. contributed equally to this work. K.Y. conceived and supervised the project. L.W., X.J., J.Y., S.R., and Y.Y. performed the experiments. L.W., X.J., L.X., K.W., and K.Y. analysed the data. X.J., L.W., D.L.L.‐A., L.H.‐E., H.L., and K.Y. wrote the paper with input from all the authors.

## Supporting information


**Figure S1** Assessment of P‐removal ability of CC‐4533 with 31 mg/L P‐supply.
**Figure S2** Gene ontology (GO) enrichment analysis of significantly up‐regulated genes in *Crptc1* under phosphorus (P)‐deficient conditions.
**Figure S3** Growth of CC‐4533 and the SPAO23 line in the TAP and TA media.
**Figure S4** SPAO23 showed the highest polyP accumulation and the slowest relative polyP decline upon P deprivation.
**Figure S5** Algal fertilizer experiments of CC‐4533 and SPAO23 strains.
**Table S1** Summary of phosphorus (P)‐removal setups and P‐removal efficiencies of bacterial PAOs and algal PAOs.
**Table S2** Phosphorus (P) concentrations measured in several representative plants.
**Table S3** Elemental analysis of CC‐4533 and SPAO23.
**Table S4** Primers used in this study.

## Data Availability

All sequencing reads have been submitted to the NCBI database under accession number PRJNA831360. Sequence data from this article can be found on the websites of Phytozome (https://phytozome‐next.jgi.doe.gov) under the following accession numbers: PTC1 (Cre06.g251650), PSR1 (Cre12.g495100) and PTB2 (Cre07.g325741). Processed data required to reproduce the figures in this study are available on Figshare (https://doi.org/10.6084/m9.figshare.15020289).

## References

[pbi14040-bib-0001] Abeysiriwardana‐Arachchige, I.S.A. , Delanka‐Pedige, H.M.K. , Munasinghe‐Arachchige, S.P. and Nirmalakhandan, N. (2021) Techno‐economic optimization of phosphorous recovery in an algal‐based sewage treatment system. Bioresour. Technol. 332, 125128. 33853026 10.1016/j.biortech.2021.125128

[pbi14040-bib-0002] Aksoy, M. , Pootakham, W. and Grossman, A.R. (2014) Critical function of a Chlamydomonas reinhardtii putative polyphosphate polymerase subunit during nutrient deprivation. Plant Cell 26, 4214–4229. 25281687 10.1105/tpc.114.129270 PMC4247568

[pbi14040-bib-0003] Aschar‐Sobbi, R. , Abramov, A.Y. , Diao, C. , Kargacin, M.E. , Kargacin, G.J. , French, R.J. and Pavlov, E. (2008) High sensitivity, quantitative measurements of polyphosphate using a new DAPI‐based approach. J. Fluoresc. 18, 859–866. 18210191 10.1007/s10895-008-0315-4

[pbi14040-bib-0004] Bajhaiya, A.K. , Dean, A.P. , Zeef, L.A.H. , Webster, R.E. and Pittman, J.K. (2016) PSR1 is a global transcriptional regulator of phosphorus deficiency responses and carbon storage metabolism in Chlamydomonas reinhardtii. Plant Physiol. 170, 1216–1234. 26704642 10.1104/pp.15.01907 PMC4775146

[pbi14040-bib-0005] Chang, C.‐W. , Moseley, J.L. , Wykoff, D. and Grossman, A.R. (2005) The LPB1 gene is important for acclimation of Chlamydomonas reinhardtii to phosphorus and sulfur deprivation. Plant Physiol. 138, 319–329. 15849300 10.1104/pp.105.059550 PMC1104186

[pbi14040-bib-0006] Cogan, E.B. , Birrell, G.B. and Griffith, O.H. (1999) A robotics‐based automated assay for inorganic and organic phosphates. Anal. Biochem. 271, 29–35. 10361001 10.1006/abio.1999.4100

[pbi14040-bib-0007] Cong, W.‐F. , Suriyagoda, L.D.B. and Lambers, H. (2020) Tightening the phosphorus cycle through phosphorus‐efficient crop genotypes. Trends Plant Sci. 25, 967–975. 32414603 10.1016/j.tplants.2020.04.013

[pbi14040-bib-0008] Cordell, D. and White, S. (2014) Life's Bottleneck: Sustaining the World's Phosphorus for a Food Secure Future. Annu. Rev. Env. Resour. 39, 161–188.

[pbi14040-bib-0009] Cordell, D. , Drangert, J.‐O. and White, S. (2009) The story of phosphorus: Global food security and food for thought. Glob. Environ. Change 19, 292–305.

[pbi14040-bib-0010] Elser, J. and Bennett, E. (2011) A broken biogeochemical cycle. Nature 478, 29–31. 21979027 10.1038/478029a

[pbi14040-bib-0011] Gilbert, N. (2009) Environment: The disappearing nutrient. Nature 461, 716–718. 19812648 10.1038/461716a

[pbi14040-bib-0012] Harris, E.H. (1989) The Chlamydomonas Sourcebook. San Diego: Elsevier.

[pbi14040-bib-0013] Jia, X. , Wang, L. , Zeng, H. and Yi, K. (2021) Insights of intracellular/intercellular phosphate transport and signaling in unicellular green algae and multicellular land plants. New Phytol. 232, 1566–1571. 34482553 10.1111/nph.17716

[pbi14040-bib-0014] Jia, X. , Wang, L. , Nussaume, L. and Yi, K. (2023) Cracking the code of plant central phosphate signaling. Trends Plant Sci. 28, 267–270. 36588035 10.1016/j.tplants.2022.12.008

[pbi14040-bib-0015] Li, K. , Liu, Q. , Fang, F. , Luo, R. , Lu, Q. , Zhou, W. , Huo, S. *et al*. (2019a) Microalgae‐based wastewater treatment for nutrients recovery: A review. Bioresour. Technol. 291, 121934. 31395401 10.1016/j.biortech.2019.121934

[pbi14040-bib-0016] Li, X. , Patena, W. , Fauser, F. , Jinkerson, R.E. , Saroussi, S. , Meyer, M.T. , Ivanova, N. *et al*. (2019b) A genome‐wide algal mutant library and functional screen identifies genes required for eukaryotic photosynthesis. Nat. Genet. 51, 627–635. 30886426 10.1038/s41588-019-0370-6 PMC6636631

[pbi14040-bib-0017] Liu, X. , Sheng, H. , Jiang, S. , Yuan, Z. , Zhang, C. and Elser, J.J. (2016) Intensification of phosphorus cycling in China since the 1600s. Proc. Natl. Acad. Sci. USA 113, 2609–2614. 26903638 10.1073/pnas.1519554113 PMC4790974

[pbi14040-bib-0018] MacDonald, G.K. , Bennett, E.M. , Potter, P.A. and Ramankutty, N. (2011) Agronomic phosphorus imbalances across the world's croplands. Proc. Natl. Acad. Sci. USA 108, 3086–3091. 21282605 10.1073/pnas.1010808108 PMC3041096

[pbi14040-bib-0019] Mekonnen, M.M. and Hoekstra, A.Y. (2018) Global anthropogenic phosphorus loads to freshwater and associated grey water footprints and water pollution levels: A high‐resolution global study. Water Resour. Res. 54, 345–358.

[pbi14040-bib-0020] Mohsenpour, S.F. , Hennige, S. , Willoughby, N. , Adeloye, A. and Gutierrez, T. (2021) Integrating micro‐algae into wastewater treatment: A review. Sci. Total Environ. 752, 142168. 33207512 10.1016/j.scitotenv.2020.142168

[pbi14040-bib-0021] Moseley, J.L. , Chang, C.‐W. and Grossman, A.R. (2006) Genome‐based approaches to understanding phosphorus deprivation responses and PSR1 control in Chlamydomonas reinhardtii. Eukaryot. Cell 5, 26–44. 16400166 10.1128/EC.5.1.26-44.2006 PMC1360252

[pbi14040-bib-0022] Nie, X. , Mubashar, M. , Zhang, S. , Qin, Y. and Zhang, X. (2020) Current progress, challenges and perspectives in microalgae‐based nutrient removal for aquaculture waste: A comprehensive review. J. Clean. Prod. 277, 124209.

[pbi14040-bib-0023] Nosheen, S. , Ajmal, I. and Song, Y. (2021) Microbes as biofertilizers, a potential approach for sustainable crop production. Sustainability 13, 1868.

[pbi14040-bib-0024] Peñuelas, J. and Sardans, J. (2022) The global nitrogen‐phosphorus imbalance. Science 375, 266–267. 35050668 10.1126/science.abl4827

[pbi14040-bib-0025] Powell, N. , Shilton, A. , Chisti, Y. and Pratt, S. (2009) Towards a luxury uptake process via microalgae – Defining the polyphosphate dynamics. Water Res. 43, 4207–4213. 19616819 10.1016/j.watres.2009.06.011

[pbi14040-bib-0026] Procházková, G. , Brányiková, I. , Zachleder, V. and Brányik, T. (2014) Effect of nutrient supply status on biomass composition of eukaryotic green microalgae. J. Appl. Phycol. 26, 1359–1377.

[pbi14040-bib-0027] Qin, L. , Gao, M. , Zhang, M. , Feng, L. , Liu, Q. and Zhang, G. (2020) Application of encapsulated algae into MBR for high‐ammonia nitrogen wastewater treatment and biofouling control. Water Res. 187, 116430. 33011566 10.1016/j.watres.2020.116430

[pbi14040-bib-0028] Quisel, J.D. , Wykoff, D.D. and Grossman, A.R. (1996) Biochemical characterization of the extracellular phosphatases produced by phosphorus‐deprived Chlamydomonas reinhardtii. Plant Physiol. 111, 839–848. 8754684 10.1104/pp.111.3.839 PMC157902

[pbi14040-bib-0029] Reich, P.B. and Oleksyn, J. (2004) Global patterns of plant leaf N and P in relation to temperature and latitude. Proc. Natl. Acad. Sci. USA 101, 11001–11006. 15213326 10.1073/pnas.0403588101 PMC503733

[pbi14040-bib-0030] Rubio, V. , Linhares, F. , Solano, R. , Martín, A.C. , Iglesias, J. , Leyva, A. and Paz‐Ares, J. (2001) A conserved MYB transcription factor involved in phosphate starvation signaling both in vascular plants and in unicellular algae. Genes Dev. 15, 2122–2133. 11511543 10.1101/gad.204401 PMC312755

[pbi14040-bib-0031] Schindler, D.W. , Carpenter, S.R. , Chapra, S.C. , Hecky, R.E. and Orihel, D.M. (2016) Reducing phosphorus to curb lake eutrophication is a success. Environ. Sci. Technol. 50, 8923–8929. 27494041 10.1021/acs.est.6b02204

[pbi14040-bib-0032] Shilton, A.N. , Powell, N. and Guieysse, B. (2012) Plant based phosphorus recovery from wastewater via algae and macrophytes. Curr. Opin. Biotechnol. 23, 884–889. 22889679 10.1016/j.copbio.2012.07.002

[pbi14040-bib-0033] Shimogawara, K. , Fujiwara, S. , Grossman, A. and Usuda, H. (1998) High‐efficiency transformation of Chlamydomonas reinhardtii by electroporation. Genetics 148, 1821–1828. 9560396 10.1093/genetics/148.4.1821 PMC1460073

[pbi14040-bib-0034] Solovchenko, A. , Verschoor, A.M. , Jablonowski, N.D. and Nedbal, L. (2016) Phosphorus from wastewater to crops: An alternative path involving microalgae. Biotechnol. Adv. 34, 550–564. 26795876 10.1016/j.biotechadv.2016.01.002

[pbi14040-bib-0035] Sugiura, S.H. (2018) Phosphorus, aquaculture, and the environment. Rev. Fish. Sci. Aquac. 26, 515–521.

[pbi14040-bib-0036] de Vasconcelos, V.M. , de Morais, E.R.C. , Faustino, S.J.B. , Hernandez, M.C.R. , da Gaudêncio, H.R.S.C. , de Melo, R.R. and Bessa Junior, A.P. (2021) Floating aquatic macrophytes for the treatment of aquaculture effluents. Environ. Sci. Pollut. Res. 28, 2600–2607. 10.1007/s11356-020-11308-8 33125679

[pbi14040-bib-0037] Wang, L. , Xiao, L. , Yang, H. , Chen, G. , Zeng, H. , Zhao, H. and Zhu, Y. (2020) Genome‐wide identification, expression profiling, and evolution of phosphate transporter gene family in green algae. Front. Genet. 11, 1170. 10.3389/fgene.2020.590947 PMC7578391 33133172

[pbi14040-bib-0038] Wang, L. , Jia, X. , Zhang, Y. , Xu, L. , Menand, B. , Zhao, H. , Zeng, H. *et al*. (2021) Loss of two families of SPX domain‐containing proteins required for vacuolar polyphosphate accumulation coincides with the transition to phosphate storage in green plants. Mol. Plant 14, 838–846. 33515767 10.1016/j.molp.2021.01.015

[pbi14040-bib-0039] Werner, T.P. , Amrhein, N. and Freimoser, F.M. (2005) Novel method for the quantification of inorganic polyphosphate (iPoP) in Saccharomyces cerevisiae shows dependence of iPoP content on the growth phase. Arch. Microbiol. 184, 129–136. 16184370 10.1007/s00203-005-0031-2

[pbi14040-bib-0040] Win, T.T. , Barone, G.D. , Secundo, F. and Fu, P. (2018) Algal biofertilizers and plant growth stimulants for sustainable agriculture. Ind. Biotechnol. 14, 203–211.

[pbi14040-bib-0041] Withers, P.J.A. (2019) Closing the phosphorus cycle. Nat. Sustain. 2, 1001–1002.

[pbi14040-bib-0042] Wykoff, D.D. , Grossman, A.R. , Weeks, D.P. , Usuda, H. and Shimogawara, K. (1999) Psr1, a nuclear localized protein that regulates phosphorus metabolism in Chlamydomonas. Proc. Natl. Acad. Sci. USA 96, 15336–15341. 10611385 10.1073/pnas.96.26.15336 PMC24820

[pbi14040-bib-0043] Xu, Y. , Wu, Y. , Esquivel‐Elizondo, S. , Dolfing, J. and Rittmann, B.E. (2020) Using microbial aggregates to entrap aqueous phosphorus. Trends Biotechnol. 38, 1292–1303. 32307119 10.1016/j.tibtech.2020.03.012

[pbi14040-bib-0044] Yang, S.‐Y. , Huang, T.‐K. , Kuo, H.‐F. and Chiou, T.‐J. (2017) Role of vacuoles in phosphorus storage and remobilization. J. Exp. Bot. 68, 3045–3055. 28077447 10.1093/jxb/erw481

[pbi14040-bib-0045] Yao, M. , Elling, F.J. , Jones, C. , Nomosatryo, S. , Long, C.P. , Crowe, S.A. , Antoniewicz, M.R. *et al*. (2016) Heterotrophic bacteria from an extremely phosphate‐poor lake have conditionally reduced phosphorus demand and utilize diverse sources of phosphorus. Environ. Microbiol. 18, 656–667. 26415900 10.1111/1462-2920.13063 PMC5872838

[pbi14040-bib-0046] Zhang, J. and Elser, J.J. (2017) Carbon:nitrogen:phosphorus stoichiometry in fungi: A meta‐analysis. Front. Microbiol. 8, 1281. 28751879 10.3389/fmicb.2017.01281 PMC5508194

[pbi14040-bib-0047] Zhang, W. , Willows, R.D. , Deng, R. , Li, Z. , Li, M. , Wang, Y. , Guo, Y. *et al*. (2021) Bilin‐dependent regulation of chlorophyll biosynthesis by GUN4. Proc. Natl. Acad. Sci. USA 118, e2104443118. 33975960 10.1073/pnas.2104443118 PMC8158021

[pbi14040-bib-0048] Zhang, Y. , Wang, L. , Guo, Z. , Xu, L. , Zhao, H. , Zhao, P. , Ma, C. *et al*. (2022) Revealing the underlying molecular basis of phosphorus recycling in the green manure crop Astragalus sinicus. J. Clean. Prod. 341, 130924.

