# Supplementary material for: Engineering microalgae for water phosphorus recovery to close the phosphorus cycle
Source: Plant Biotechnol J. 2023 Mar 15;21(7):1373–82. doi: 10.1111/pbi.14040 (PMC10281605; doi:10.1111/pbi.14040)
Supplement: Supplementary file 1 — Figure S1 Assessment of P‐removal ability of CC‐4533 with 31 mg/L P‐supply. Figure S2 Gene ontology (GO) enrichment analysis of significantly up‐regulated genes in Crptc1 under phosphorus (P)‐deficient conditions. Figure S3 Growth of CC‐4533 and the SPAO23 line in the TAP and TA media. Figure S4 SPAO23 showed the highest polyP accumulation and the slowest relative polyP decline upon P deprivation. Figure S5 Algal fertilizer experiments of CC‐4533 and SPAO23 strains. Table S1 Summary of phosphorus (P)‐removal setups and P‐removal efficiencies of bacterial PAOs and algal PAOs. Table S2 Phosphorus (P) concentrations measured in several representative plants. Table S3 Elemental analysis of CC‐4533 and SPAO23. Table S4 Primers used in this study. [file PBI-21-1373-s001.pdf]

Supporting Information for

Engineering microalgae for water phosphorus recovery to close the phosphorus cycle

Correspondence to: yikeke@gmail.com (K.Y.); jiaxq.nju@gmail.com (X.J.)

This PDF file includes:

Figure S1 to S5

Table S1 to S4

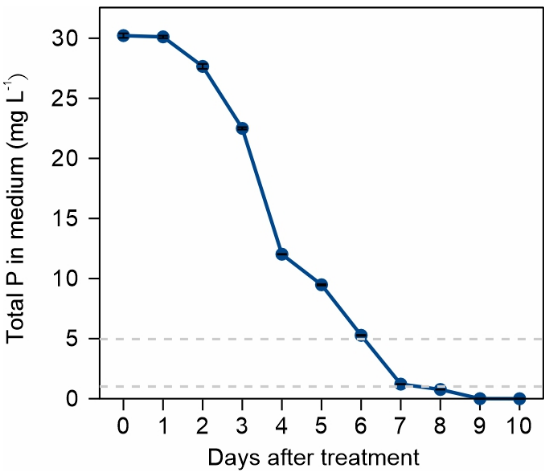

Figure S1. Assessment of P-removal ability of CC-4533 with 31 mg L^{-1} P-supply.

Grey lines on 1 and 5 mg L^{-1} show the first-level and third-level water quality threshold values, respectively, according to the discharge standard of pollutants for municipal wastewater treatment plants in China (GB18918-2002). Error bars indicate SE.

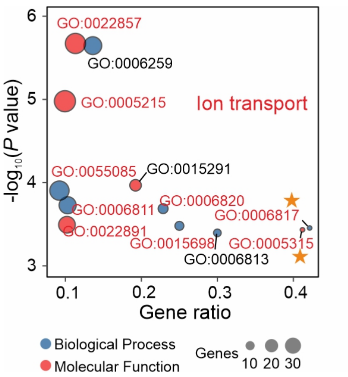

Category	ID	Term	Gene_ratio	pvalue	adj_pval
Biological Process	GO:0006259	DNA metabolic process	0.136	2.00E-06	0.00049
	GO:0006820	anion transport	0.229	0.00026	0.02
	GO:0006811	ion transport	0.103	0.00024	0.02
	GO:0055085	transmembrane transport	0.092	0.00017	0.02
	GO:0006813	potassium ion transport	0.300	0.00046	0.024
★	GO:0006817	phosphate ion transport	0.417	0.00041	0.024
	GO:0015698	inorganic anion transport	0.250	0.00039	0.024
Molecular Function	GO:0022857	transmembrane transporter activity	0.113	1.90E-06	0.0006
	GO:0005215	transporter activity	0.099	7.40E-06	0.0012
	GO:0015291	secondary active transmembrane transporter activity	0.192	0.00015	0.016
★	GO:0005315	inorganic phosphate transmembrane transporter activity	0.417	0.00041	0.027
	GO:0022891	substrate-specific transmembrane transporter activity	0.102	0.00038	0.027

Figure S2. Gene ontology (GO) enrichment analysis of significantly up-regulated genes in *Crptc1* under phosphorus (P)-deficient conditions.

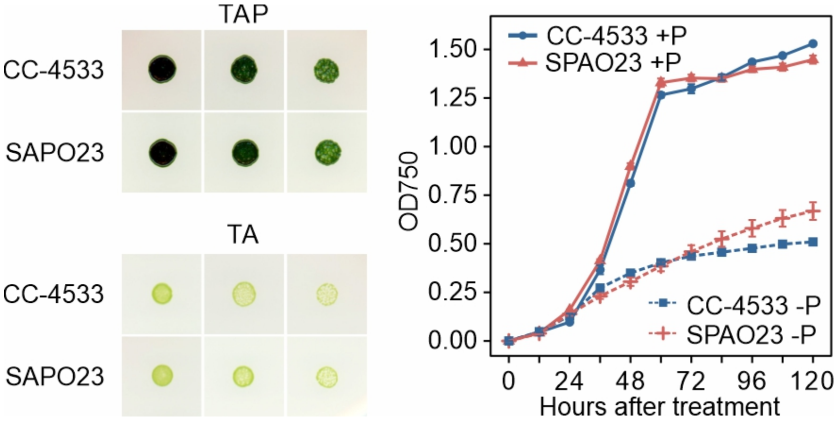

Figure S3. Growth of CC-4533 and the SPAO23 line in the TAP and TA media.

Left panel, colonies from left to right represent a series of dilutions. The panel at the right shows the growth curves of CC-4533 and the SPAO23 line under 31 mg L^{-1} P-supply (+P) and Pi-deprivation (-P) conditions.

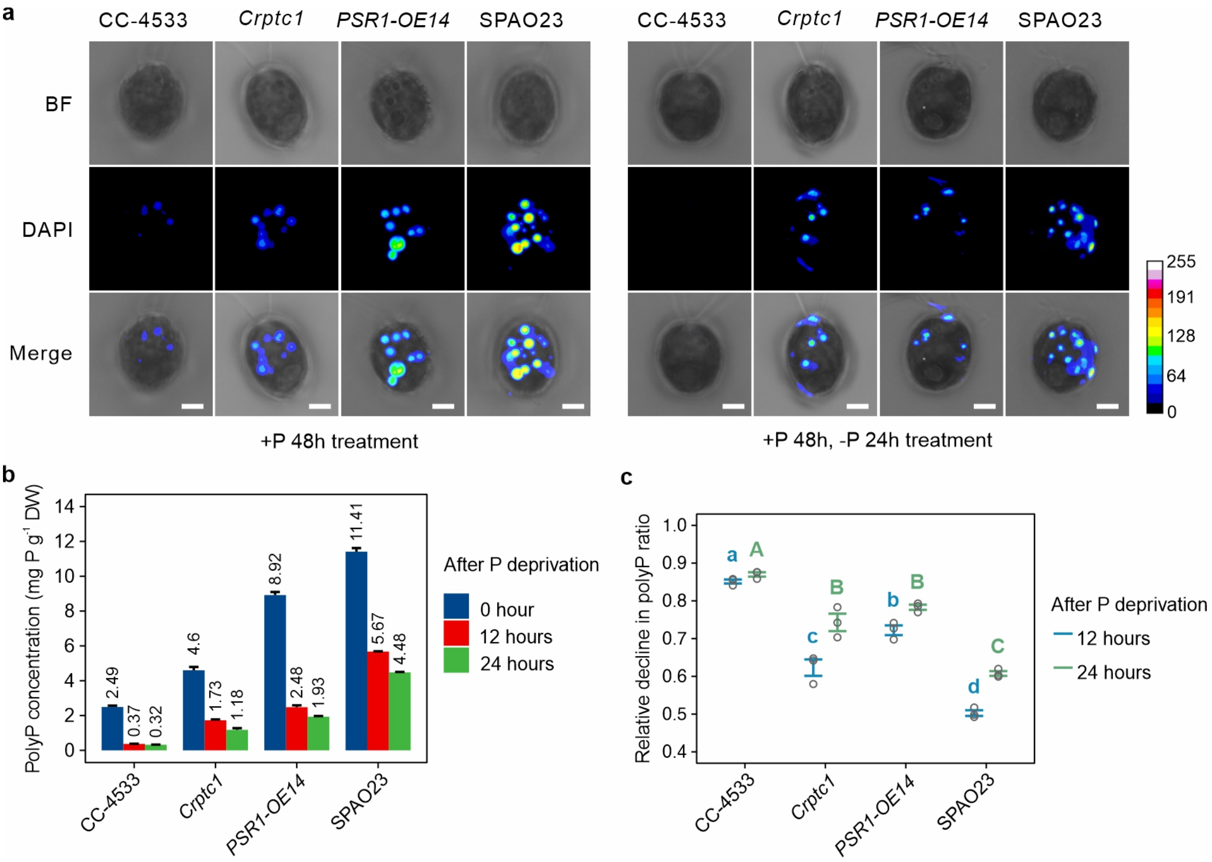

Figure S4. SPAO23 showed the highest polyP accumulation and the slowest relative polyP decline upon P deprivation.

a) Representative pseudo-color images of cellular polyP stained with DAPI. Bar, 5 μm. Experiments were repeated three times with similar results. **b)** PolyP concentrations of CC-4533, the *Crptc1* mutant, *PSR1-OE14* line, and SPAO23 line after phosphorus (P) deprivation. Error bars indicate SE.**c)** The relative decline in the polyP ratio of different strains under P deprivation. Differences were tested by ANOVA using the LSD method with a Bonferroni correction at $\alpha = 0.05$. Letters with the same color (light blue or dark blue) refer to the test for the same group. Means with the same letters were not significantly different.

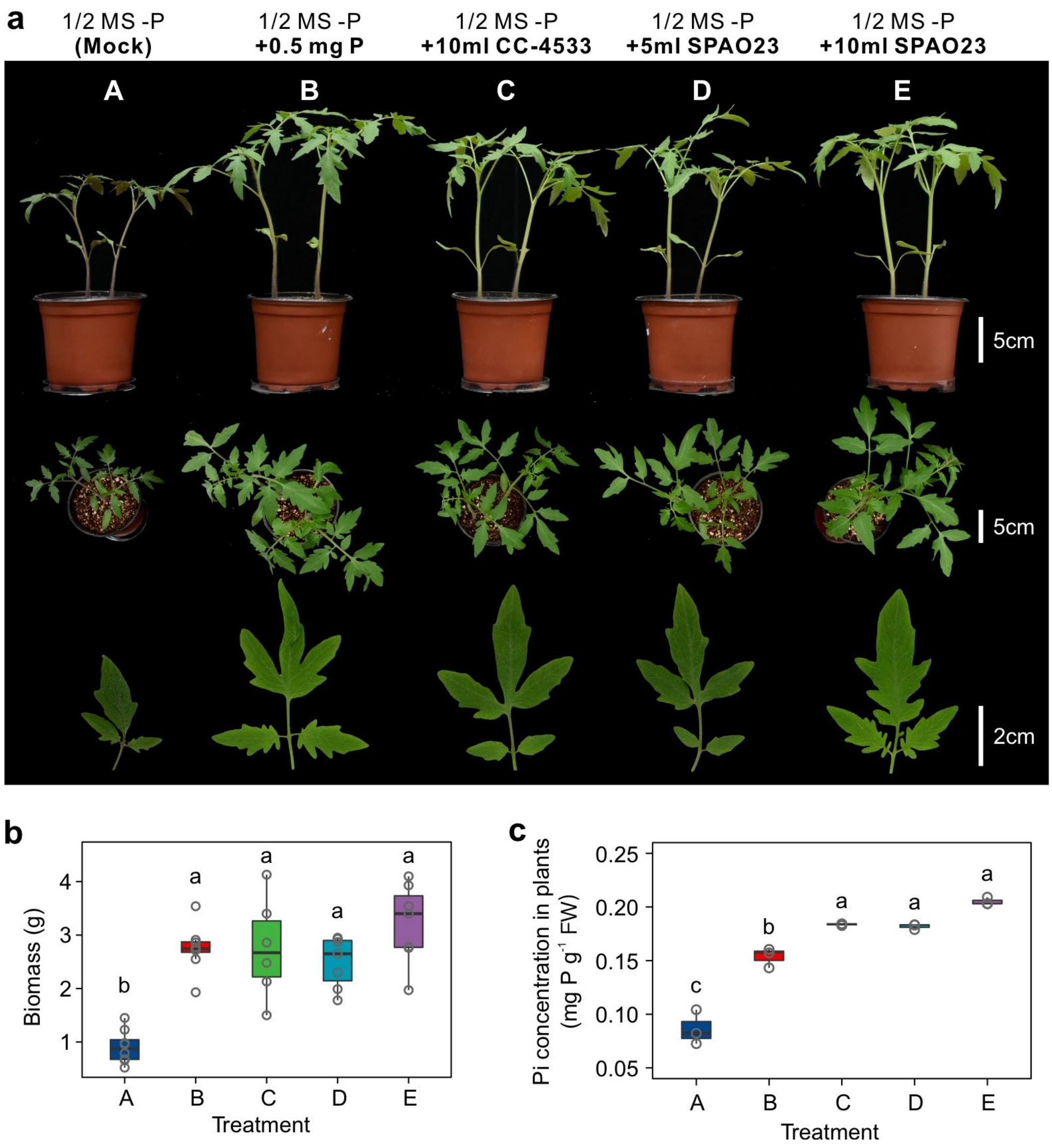

Figure S5. Algal fertilizer experiments of CC-4533 and SPAO23 strains.

a) Plant architecture of each treatment. The panels are ordered as follows: front view, top view of shoots, and the youngest fully-developed leaves. **b)** Biomass of plants collected from each treatment; at least seven individuals for each treatment. **c)** Inorganic phosphorus (Pi) concentration in plants from each treatment; three individuals for each treatment. Differences were tested by ANOVA using the LSD method with a Bonferroni correction at $\alpha = 0.05$. Means with the same letters are not significantly different. Error bars indicate SE.

Table S1. Summary of phosphorus (P)-removal setups and P-removal efficiencies of bacterial PAOs and algal PAOs.

Bacterial PAOs	Initial P concentration (mg L^{-1})	Initial inoculum	Working volume (L)	Time (h)	Removal rate (%)	Relative P removal (mg L^{-1} h^{-1})	Ref.
Acinetobacter calcoaceticus	38	4%	0.1	5	48	3.68	Ref [1]
Alcaligenes denitrificans	38	4%	0.1	5	36	2.76	
Aeromonas hydrophila	38	4%	0.1	5	23	1.72	
Bacillus cereus	38	4%	0.1	5	36	2.74	
Moraxella phenylpyruvica	38	4%	0.1	5	42	3.16	
Pseudomonas acidovorans	38	4%	0.1	5	23	1.78	
Pseudomonas fluorescens	38	4%	0.1	5	54	4.12	
Pseudomonas mendocina	38	4%	0.1	5	52	3.92	
Pseudomonas testesteroni	38	4%	0.1	5	35	2.66	
Staphylococcus epidermidis	38	4%	0.1	5	27	2.08	
Acinetobacter junii	25	5%	ND*	12	92	1.92	Ref [2]
Acinetobacter junii	70	5%	ND*	12	66	3.84	
**Algal PAOs**	**Initial P concentration (mg L^{-1})**	**Initial inoculum**	**Working volume (L)**	**Time (d)**	**Removal rate (%)**	**Relative removal (mg L^{-1} d^{-1})**	**Ref.**
Platymonas subcordiformis	8.8	5%	0.3	14	99	0.63	Ref [3]
Chlorella sp.	19.11	3.5%	0.1	14	82.36	1.12	Ref [4]
Chlamydomonas sp.	19.11	3.5%	0.1	14	100	1.95	
Dunaliella sp.	7	0.23 g/L	1.2	14	94.02	0.87	Ref [5]
Nannochloropsis sp.	7	0.23 g/L	1.2	14	97.12	0.78	
Tetraselmis sp.	7	0.23 g/L	1.2	14	95.62	1.49	
Scenedesmus obliquus	8.82	0.19 g/L	0.5	14	100	0.63	Ref [6]
Ankistrodesmus falcatus	8.82	0.2 g/L	0.5	14	98.52	0.62	
Chlorella sorokiniana	8.82	0.22 g/L	0.5	14	100	0.63	
Chlorella sp. GD	6.8	0.3 g/L	1	7	90	0.96	Ref [7]
Scenedesmus sp.	3.87	0.3 g/L	0.8	24	95	0.15	Ref [8]
C. reinhardtii (CC-4533)	31	1%	0.05	9	99.9	3.33	This study
Crptc1	31	1%	0.05	6	100	5	
PSR1-OE14	31	1%	0.05	3	100	10	
SPAO23	31	1%	0.05	2.5	100	12	

* ND, no data mentioned in their papers.

References:

[1] M. Sidat, F. Bux, H. Kasan, *Water SA* 1999, *25*, 175.

[2] Y.-H. Han, T. Fu, S.-S. Wang, H.-T. Yu, P. Xiang, W.-X. Zhang, D.-L. Chen, M. Li, *3*

Biotech 2018, *8*, 313.
[3] Z. Guo, Y. Liu, H. Guo, S. Yan, J. Mu, *J. Environ. Sci.* 2013, *25*, S85.
[4] S. Rasoul-Amini, N. Montazeri-Najafabady, S. Shaker, A. Safari, A. Kazemi, P. Mousavi, M. A. Mobasher, Y. Ghasemi, *Biocatal. Agric. Biotechnol.* 2014, *3*, 126.
[5] M. Sacristán de Alva, V. M. Luna Pabello, M. T. Orta Ledesma, M. J. Cruz Gómez, *Algal Res.* 2018, *34*, 97.
[6] F. A. Ansari, P. Singh, A. Guldhe, F. Bux, *Algal Res.* 2017, *21*, 169.
[7] C.-M. Kuo, J.-F. Jian, T.-H. Lin, Y.-B. Chang, X.-H. Wan, J.-T. Lai, J.-S. Chang, C.-S. Lin, *Bioresour. Technol.* 2016, *221*, 241.
[8] L. G. Cardoso, J. H. Duarte, B. B. Andrade, P. V. F. Lemos, J. A. V. Costa, J. I. Druzian, F. A. Chinalia, *Aquaculture* 2020, *525*, 735272.

Table S2. Phosphorus (P) concentrations measured in several representative plants.

Clade	Species	Strain	P concentration ($mg\ g^{-1}$ DW)
Monocots	*Zea mays*	-	1.36
Monocots	*Eichhornia crassipes* *	-	6.92
Monocots	*Lemna minor* *	-	7.43
Monocots	*Spirodela polyrrhiza* *	-	2.79
Eudicots	*Arabidopsis thaliana* *	Col-0	1.98
Eudicots	*Populus cathayana*	-	1.73
Eudicots	*Salix babylonica*	-	2.23
Eudicots	*Prunus cerasifera*	-	2.03
Eudicots	*Styphnolobium japonicum*	-	4.43
Eudicots	*Prunus davidiana*	-	1.57
Eudicots	*Vitis vinifera*	-	2.28
Gymnosperms	*Platycladus orientalis*	-	1.72
Gymnosperms	*Pinus massoniana*	-	1.01
Gymnosperms	*Cycas revoluta*	-	1.39
Gymnosperms	*Ginkgo biloba*	-	2.54
Liverworts	*Marchantia polymorpha* *	Tak-1	5.69
Streptophyte algae	*Klebsormidium sp.* *	FACHB-1489	7.27
Streptophyte algae	*Spirogyra gracilis* *	FACHB-354	7.86
Chlorophyta	*Pediastrum duplex* *	FACHB-2909	10.89
Chlorophyta	*Scenedesmus quadricauda* *	FACHB-508	16.67
Chlorophyta	*Scenedesmus quadricauda* *	FACHB-1297	18.02
Chlorophyta	*Scenedesmus spinosus* *	FACHB-1268	21.00
Chlorophyta	*Chlamydomonas reinhardtii* *	CC-4533	16.51 - 20.0**
Chlorophyta	*Chlamydomonas reinhardtii* *	*Crptc1*	22.56
Chlorophyta	*Chlamydomonas reinhardtii* *	*PSR1-OE14*	32.32
Chlorophyta	*Chlamydomonas reinhardtii* *	SPAO23	37.49 - 68.8**

* Plants cultured with non-limiting nutrients. Strains named by the prefix FACHB were collected from the Institute of Hydrobiology, Chinese Academy of Sciences, Wuhan, China. Other samples were collected from plants grown in Chinese Academy of Agricultural Sciences (CAAS), Haidian District, Beijing, 100081, China. DW, dry weight.

** To test the possible maximum P capacity, CC-4533 and SPAO23 strains were treated with a modified TAP medium which is with three times more total P content than the normal TAP medium. The total P concentration can be up to 20.0 and 68.8 mg/g DW in CC-4533 and SPAO23 strains, respectively.

Table S3. Elemental analysis of CC-4533 and SPAO23.

Elements (ppm)	CC-4533	SPAO23
Ca	4.199±0.158	7.068±0.211**
Cu	0.019±0.007	0.017±0.004
Fe	0.453±0.062	0.464±0.106
K	3.308±0.124	3.796±0.092
Mg	4.366±0.408	6.247±0.274**
Mn	0.216±0.023	0.204±0.008
Na	0.706±0.051	0.646±0.055
S	5.669±0.480	4.804±0.186
Ti	4.518±0.804	3.313±1.032
Zn	0.064±0.007	0.050±0.006

Table S4. Primers used in this study.

Primer names	Sequence (5'-3')	Use of primers
PSR1.F	GGAATTCCATATGTCGCTATGCAACGATCTACG	Over-expression line of *PSR1*
PSR1.R	CCGGATATCGCTGCCGTGAACAGTACAAA	Over-expression line of *PSR1*
PTB2.QF	AGACGGCTGAACAGTGCTAC	Quantitative RT-PCR of *PTB2*
PTB2.QR	CGTGGAGACCCATATGACCG	Quantitative RT-PCR of *PTB2*
CBLP.QF	CTTCTCGCCCATGACCAC	Quantitative RT-PCR of *CBLP*
CBLP.QR	CCCACCAGGTTGTTCTTCAG	Quantitative RT-PCR of *CBLP*
PSR1.QF	ACAGCAGCAACAAGAGCAAC	Quantitative RT-PCR of *PSR1*
PSR1.QR	CGAAATCACCGAAGTCAAAG	Quantitative RT-PCR of *PSR1*
